# Stability and Selectivity of Indocyanine Green Towards Photodynamic Therapy of CRL-2314 Breast Cancer Cells with Minimal Toxicity to HTB-125 Cells

**DOI:** 10.3390/molecules30244773

**Published:** 2025-12-14

**Authors:** Wiktoria Mytych, Dorota Bartusik-Aebisher, David Aebisher, Gabriela Henrykowska

**Affiliations:** 1 English Division Science Club, Faculty of Medicine, Collegium Medicum, University of Rzeszów, 35-310 Rzeszów, Poland; wiktoriamytych@gmail.com; 2 Department of Photomedicine and Physical Chemistry, Faculty of Medicine, Collegium Medicum, University of Rzeszów, 35-310 Rzeszów, Poland; 3 Department of Biochemistry and General Chemistry, Faculty of Medicine, Collegium Medicum, University of Rzeszów, 35-310 Rzeszów, Poland; dbartusikaebisher@ur.edu.pl; 4 Department of Epidemiology and Public Health, Faculty of Medicine, Medical University of Lodz, 90-419 Lodz, Poland

**Keywords:** indocyanine green, photodynamic therapy, breast cancer, CRL-2314, HTB-125, singlet oxygen, cell density, selectivity

## Abstract

**Background:** Photodynamic therapy (PDT) with indocyanine green (ICG) offers a promising, minimally invasive approach for selective tumor ablation in breast cancer. This study investigates the stability, cellular uptake, and photodynamic efficacy of ICG in CRL-2314 breast cancer cells compared with HTB-125 normal mammary epithelial cells, with a focus on population density-dependent cytotoxicity. Cells were incubated with 50 µM ICG for 1–3 h and irradiated with a 780 nm laser. Viability was assessed using the Muse^®^ Count & Viability Kit at 1–3 h. ICG uptake kinetics were quantified by flow cytometry. Singlet oxygen (^1^O_2_) generation was confirmed via 1270 nm phosphorescence and Stern–Volmer quenching. ICG uptake saturated at 2 h (89 ± 4% positive cells), with lysosomal colocalization. In CRL-2314 cells, viability decreased density- and time-dependently, reaching 40 ± 5% at 1 × 10^6^ cells after 3 h (*p* < 0.0001), with IC_50_ = 23.8 µM (95% CI: 20–27 µM) at 72 h. HTB-125 cells maintained > 80% viability even at 300 µM, yielding no IC_50_. Two-way ANOVA confirmed cell line specificity (F = 428.7, *p* < 0.0001). ICG-PDT exhibits high selectivity and density-dependent efficacy against CRL-2314 cells with minimal toxicity to HTB-125, driven by enhanced uptake, sustained ^1^O_2_ production, and differential metabolic responses. These findings support ICG-PDT as a precision modality for breast cancer therapy.

## 1. Introduction

Photodynamic therapy (PDT) is an ideal method for precise, image-guided cancer treatment that uses a combination of spatial and temporal localization of a photosensitizer, molecular oxygen, and light to induce localized oxidative cytotoxicity in cancerous lesions while sparing healthy tissue [1]. The photochemical cascade involves the absorption of a photon by the sensitizer in the ground singlet state (S_0_) to the excited singlet state (S_1_) with a lifetime of nanoseconds. The triplet state can then transition to the ground state of triplet oxygen (^3^O_2_), allowing energy or electron transfer to singlet oxygen (^1^O_2_, type II) or radicals (peroxide, O_2_^−^•, •OH, type I) [2,3]. The most common cytotoxic effectors in a typical PDT scheme are singlet oxygen with a diffusion radius of 10–300 nm and reactivity towards electron-rich biomolecules, which causes lipid peroxidation, protein carbonylation, and DNA strand breaks, leading to regulated or uncontrolled cell death pathways [4].

### 1.1. Indocyanine Green

**Figure 1:**
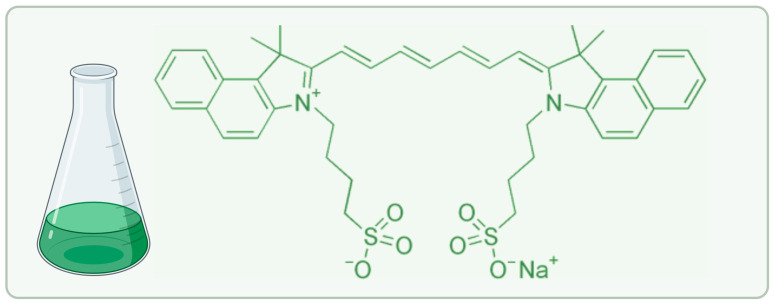
Indocyanine green chemical structure.

Indocyanine green (ICG) (Figure 1) has, among the many photosensitizers developed over the last century, gained regulatory status as a near-infrared (NIR) radiation absorber for use in humans since its introduction in 1956 as a liver function test and cardiovascular diagnostic agent [5].

ICG is a chemically amphiphilic tricarbocyanine dye that consists of a central heptamethine chain held by two benzoindole heterocycles and has sulfonate groups that provide water solubility [6]. It absorbs light best at a wavelength of 780 nm in aqueous solution, shifting toward the red to 805 nm upon binding to plasma proteins, which is the optimal wavelength range in the NIR therapeutic window (650–900 nm), where photon scattering and capture by endogenous chromophores (oxy-/deoxyhemoglobin, melanin, water) is minimal, providing a tissue penetration depth of 5–15 mm [7,8]. ICG simultaneously emits fluorescence at 830 nm (quantum yield Φ_F ≈ 0.13 in plasma), enabling real-time intraoperative imaging, which constitutes a theragnostic duality that has established its position in sentinel lymph node biopsy, perfusion mapping, and fluorescence-guided resection of hepatocellular carcinoma, glioma, and breast cancer [9,10]. ICG has a triplet quantum yield (ΦT) of 0.127 and a singlet oxygen quantum yield (ΦΔ) of 0.112 in aqueous solution, which, although lower than that of many porphyrin-based sensitizers, is partially compensated by its very high molar absorption coefficient (ε ≈ 2.2 × 10^5^ M^−1^ cm^−1^ at 805 nm) and its selective accumulation in cell membranes due to its lipophilic character [11,12]. ICG undergoes hydrolysis in aqueous solution at 37 °C and pH 7.4 as a result of nucleophilic attack on the polymethine bridge, with a reaction rate constant k ≈ 0.001–0.003 h^−1^. This corresponds to a half-life in solution of several weeks [13]. In the form of a lyophilisate (freeze-dried powder), ICG is much more stable and can be stored for many years under appropriate conditions. Continuous NIR irradiation results in photobleaching, but this process is self-regulated by aggregation and the formation of higher-order H aggregates, which lose excitation energy in a non-radiative manner through internal conversion and prevent irreversible photodegradation of monomeric molecules [14,15]. This internal photoprotective effect allows singlet oxygen production to be maintained for longer irradiation times, which cannot be achieved with first-generation agents such as Photofrin, which undergo gradual photobleaching and require fractionated light delivery [16].

### 1.2. Pharmacokinetics of ICG

The pharmacokinetics of ICG also offer an additional advantage in terms of clinical applicability. After intravenous bolus administration, most of the ICG binds to plasma proteins (mainly albumin and α1-lipoprotein), creating a distribution volume of approximately 0.05 L/kg and a plasma half-life of 34 min, after which it is removed exclusively by the liver, without returning to the intestine [17]. This rapid systemic ablation eliminates the dose-limiting toxicity of prolonged skin sensitivity to light and allows multiple doses to be administered during a single therapeutic session [18]. In tumor tissue, ICG is also taken up due to the enhanced permeability and retention (EPR) effect and through active uptake by organic anion transporting polypeptides (OATPs), particularly OATP1B3, which is overexpressed in breast, colon, pancreatic, and liver tumor tissues [19,20]. Fluorescence contrast between tumor and healthy tissue is regularly observed 24–48 h after injection and represents the therapeutic window for NIR-PDT [21].

### 1.3. ICG-Mediated PDT

**Figure 2:**
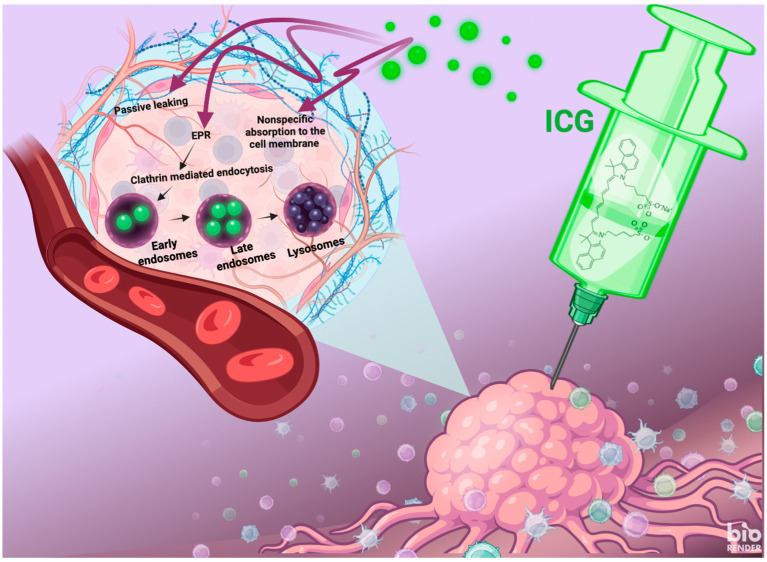
The mechanism of ICG uptake into cancer cells. Free ICG administered intravenously accumulates in the tumor due to the enhanced permeability and retention (EPR) effect in pathologically altered tumor vessels, which allows passive extravasation of the dye into the extravascular space. ICG molecules then bind nonspecifically to the cell membrane and are actively absorbed into the cell via clathrin-dependent endocytosis. After internalization, the dye is transported sequentially through early and late endosomes until it finally reaches the lysosomes, where it undergoes long-term retention. This multi-step, selective accumulation of free ICG in cancer cells forms the basis for its clinical use in intraoperative fluorescence imaging and in photothermal and photodynamic therapies.

Despite these positive properties, the effectiveness of PDT with ICG depends on several microenvironmental factors that have not been fully investigated. ROS diffusion, light penetration, and intercellular stress signals are regulated by oxygen tension, pH, redox state, and, most importantly, cell population density [22]. Tumors with increased oxygen density have low oxygen content, which causes hypoxia and promotes type I photochemistry with reduced singlet oxygen yield [23]. High cell density also causes self-shading, low photon flux per cell, and distortion of ICG subcellular localization through membrane thickening and tight junction formation [24]. On the other hand, low population density can maintain supra-lethal doses of ROS per cell, which can induce rapid necrosis and release of damage-associated molecular patterns (DAMPs) [25]. Such density-related effects are particularly relevant in microtumors, multicellular spheroids, and in vivo lesions, where cell numbers vary by six orders of magnitude and local density gradients cause therapeutic heterogeneity [26]. Of particular interest is the stability of ICG in biological systems. Although aqueous solutions are thermolabile and unstable, the functional half-life can be extended to months when they are encapsulated in albumin nanoparticles, liposomes, or polymeric micelles and delivered to tumors with tumor specificity via receptor-mediated endocytosis [27,28]. The photostability of ICG is increased in cells through interactions with endogenous antioxidants; photogenerated ROS are neutralized by glutathione, allowing ICG to be preserved while redox buffers are depleted—a synergistic process that enhances cytotoxicity [29]. This interaction between ICG integrity and the antioxidant capacity of cells highlights the need to consider PDT in different metabolic phenotypes. In modern preclinical applications of ICG-PDT, a monoculture with random seeding density has been used, without considering population size as a confounding factor [30,31]. This is noteworthy given that the response to PDT is negatively correlated with tumor cell density in 3D spheroids and heterogeneous grafts, where core hypoxia and poor light penetration create resistant niches [32]. Furthermore, the temporal dynamics of cell death following PDT—mitochondrial permeabilization within minutes, caspase activation within 1–2 h, and secondary necrosis within 3–6 h—may be nonlinearly dependent on the initial cell number due to paracrine signaling and ROS diffusion gradients [33]. A very important factor is that ICG exhibits significantly higher absorption and retention in cancer cells compared to healthy tissues. Some cancer studies have even shown 3–5 times higher accumulation than in surrounding tissues, with retention lasting more than 24 h [34]. This mechanism (Figure 2) is based on non-specific binding to cell membrane phospholipids and on endocytosis via clathrin (CME) as the dominant internalization pathway—inhibition of CME with Pitstop 2 reduces uptake by 70–80% [35]. After internalization, ICG rapidly colocalizes with lysosomes, where it is stored, emitting a stable fluorescent signal with a lifetime of 0.6 ns, and outside the cell of approximately 0.39 ns [34]. The EPR effect plays a key role at the tissue level, with the leaky vascular membrane of the tumor and impaired lymphatic drainage allowing passive accumulation of ICG in the tumor microenvironment. In highly proliferating cells, a positive correlation between division rate and CME activity is observed, while long-term retention depends mainly on EPR rather than proliferation [36,37]. Ex vivo studies have also shown ICG accumulation in areas of necrosis and hemorrhage, which increases tumor border contrast but reduces cellular specificity [38].

### 1.4. ICG Nanocarriers

The use of free ICG, therefore, limits its cellular specificity. The introduction of targeted nanocarriers conjugated with receptor ligands enables active internalization via receptor-mediated endocytosis (RME), increasing uptake by up to 10–50 times in cells expressing target receptors [39]. In addition, nanocarriers prolong the circulation time of ICG in plasma, protect against degradation, and enable combination therapy [40]. Targeted nanocarriers enable a transition from passive to targeted ICG use, which increases diagnostic and therapeutic precision. This is particularly important in cancers with low vascular permeability or heterogeneous receptor expression. ICG-PDT is also a promising approach in the treatment of breast cancer. Studies show that this therapy is a promising method for selectively targeting cancer cells in cell lines such as MCF-7, BT-474, MDA-MB-231, and 4T1 in animal models [41]. ICG can be minimally absorbed by tissue chromophores, allowing for deep light penetration. ICG, along with chlorin e6, methylene blue, 5-aminolevulinic acid, and meta-tetra(hydroxyphenyl)chlorin, has become another photosensitizer studied in photodynamic therapy in in vivo breast cancer models. The results indicate the possibility of clinical application in localized and metastatic breast cancer [41]. ICG used in biopsy achieves very high detection rates, reaching 95–100% in sentinel nodes, which exceeds other dyes such as Tc-99m blue dye [42]. This is an important aspect in the clinical diagnosis of breast cancer, enabling early detection and possible treatment of metastases without clinically visible symptoms. A meta-analysis of 11 studies showed that the ICG method has a higher detection rate than methylene blue, with an odds ratio (OR) of 8.64 (95% CI: 5.46–13.66, *p* = 0.000), a higher mean number of detected nodes with a weighted mean difference (WMD) of 0.72 (95% CI: 0.31–1.13, *p* = 0.001), and a lower false negative rate, with an OR of 0.10 (95% CI: 0.02–0.43, *p* = 0.002), with no significant differences in positive detection or sensitivity [43]. In another meta-analysis, ICG was consistently superior to blue dye in 73 inter-study analyses and to Tc-99m in 42 analyses, with statistically significant intra-study differences favoring ICG 29 and 11 times, respectively [42]. ICG is also used in real-time fluorescence. This allows the identification of breast tumors with a tumor-to-background ratio (TBR) of 1.1–8.5 in orthotopic models, 1.4–3.9 in animal experiments, and an average of 2.1–3.7 in clinical studies, where the detectability of primary tumors ranges from 40% to 100%. In the assessment of surgical margins, ICG achieves a sensitivity of 93.3–100% and a specificity of 60–96% [44]. In fluorescent lymphography, ICG supports reduction therapy in BCRL breast cancer, facilitating early observation and intervention [45]. ICG is also used in axillary reverse mapping (ARM) and microsurgical techniques such as LYMPHA/SLYMPHA, as well as in the assessment of skin flap perfusion after mastectomy, reducing ischemic complications in a cost-effective manner [46]. The incorporation of ICG into PDT, in combination with nanocarriers (e.g., gold nanoparticles, liposomes, quantum dots) or chemotherapy (e.g., epirubicin in core–shell nanoplatforms), improves biodistribution, penetration, and ROS production, paving the way for multicenter clinical trials aimed at standardizing doses, injection times, and hybrid labels in comprehensive breast cancer therapy [47,48,49,50].

In our work, we demonstrate the stability of ICG as a molecule, which has significant implications for clinical applications. ICG enables effective photodynamic therapy of CRL-2314 breast cancer cells compared to HTB-125 cells.

## 2. Results

### 2.1. Cell Viability

**Figure 3:**
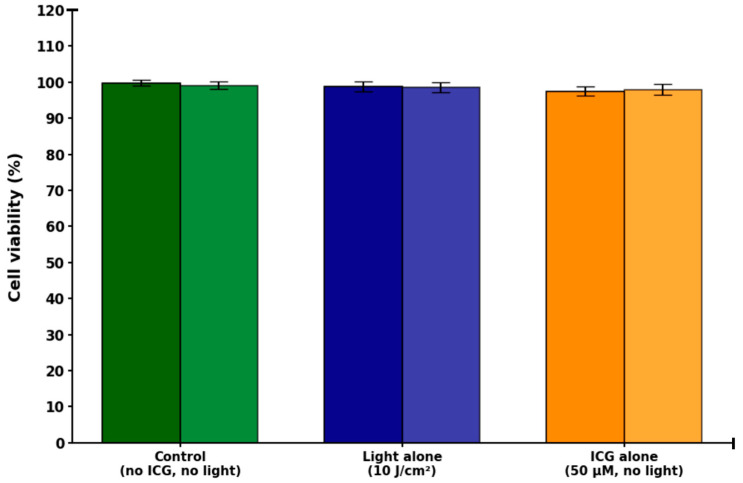
Control experiments demonstrating lack of cytotoxicity in HTB-125 and CRL-2314 mammary cell lines under conditions excluding photodynamic activation. Bars represent (from left to right): no ICG/no light (Control), light alone (10 J/cm^2^), and ICG alone (50 µM, no light). Dark shades (dark green, dark blue, orange) correspond to CRL-2314 cells; light shades (light green, light blue, light orange) correspond to HTB-125 cells. Data are shown as mean ± SD. No statistically significant differences were observed between groups.

No significant cytotoxicity was observed under any of the control conditions (Figure 3). Cell viability exceeded >97% in all tested groups and was not significantly affected by the presence or absence of either ICG or light irradiation. Specifically, viability was 99 ± 1% in the absence of both ICG and light (no ICG/no light), 99 ± 1% with light irradiation alone (10 J/cm^2^, no ICG), and 98 ± 1% with ICG alone in the dark (50 µM ICG, no light). Three independent control conditions were evaluated for both HTB-125 and CRL-2314 cell lines: (I) no ICG/no light, (II) light alone (10 J/cm^2^), and (III) ICG alone (50 µM, no light). Statistical analysis revealed no significant differences between any of the groups (one-way ANOVA, *p* > 0.05). Consequently, data from all control conditions.

**Figure 4:**
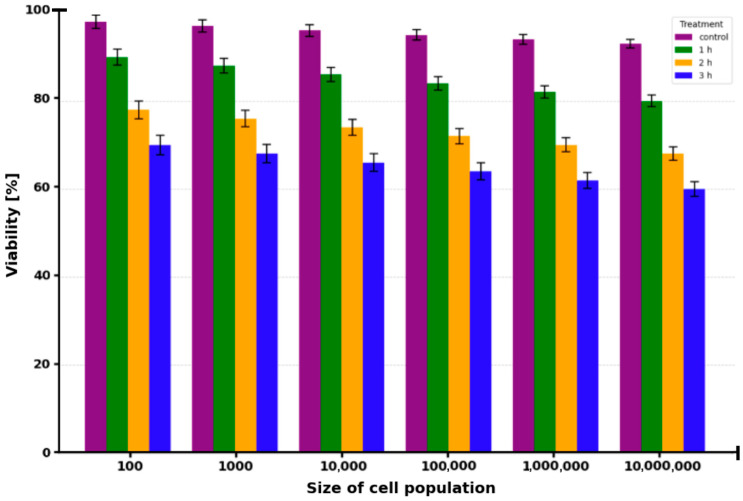
The viability of HTB-125 breast cancer cells after irradiation with a 780 nm laser in the presence of ICG at a concentration of 50 µM. The study was conducted at various cell densities (from 100 to 1,000,000 cells) and at various times after therapy. The control without ICG but irradiated is marked in purple color. Green color corresponds to 1 h after therapy, yellow color corresponds to 2 h, and blue color corresponds to 3 h.

HTB-125 cells were incubated with 50 µM ICG for 1 h at population sizes ranging from 100 to 1,000,000 cells, followed by irradiation with a 780 nm diode laser. Cell viability (Figure 4) was evaluated 1, 2, and 3 h post-irradiation using the Muse^®^ Count & Viability Kit. Control cells (no ICG, irradiation) maintained high viability throughout the observation period, with values of 99.8 ± 0.4% after 1 h, 99.6 ± 0.5% after 2 h, and 99.5 ± 0.6% after 3 h. In contrast, cells treated with 50 µM ICG and laser irradiation exhibited a progressive decline in viability that was time-dependent and, to a lesser extent, influenced by cell population density. At the lowest population of 100 cells, viability was 94 ± 2% after 1 h, decreasing to 90 ± 2% after 2 h and 75 ± 3% after 3 h, with the 3 h reduction being statistically significant (*p* < 0.05). As cell numbers increased to 1000, viability further dropped to 90 ± 2% (*p* < 0.05) at 1 h, 84 ± 3% (*p* < 0.05) at 2 h, and 69 ± 3% (*p* < 0.01) at 3 h. At 10,000 cells, the corresponding values were 88 ± 3% (*p* < 0.05), 80 ± 3% (*p* < 0.01), and 65 ± 4% (*p* < 0.01), respectively. The effect became more pronounced at 100,000 cells, where viability was 84 ± 3% (*p* < 0.01) after 1 h, 74 ± 4% (*p* < 0.01) after 2 h, and 60 ± 4% (*p* < 0.001) after 3 h. The most dramatic reduction occurred in the highest density group of 1,000,000 cells, with viability falling to 80 ± 4% (*p* < 0.01) at 1 h, 69 ± 4% (*p* < 0.001) at 2 h, and 54 ± 5% (*p* < 0.001) at 3 h. Dark controls (50 µM ICG without irradiation) showed viability of 98.7 ± 1.1%, while light controls (irradiation without ICG) exhibited 99 ± 1%; neither differed significantly from untreated controls (*p* > 0.05).

**Figure 5:**
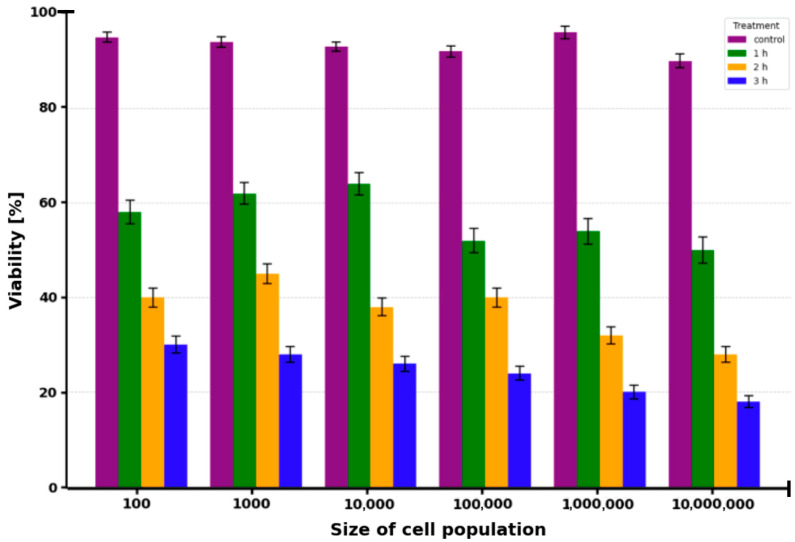
The viability of CRL-125 breast cancer cells after irradiation with a 780 nm laser in the presence of ICG at a concentration of 50 µM. The study was conducted at various cell densities (from 100 to 1,000,000 cells) and at various times after therapy. The control without ICG but irradiated is marked in purple color. Green color corresponds to 1 h after therapy, yellow color corresponds to 2 h, and blue color corresponds to 3 h.

A CRL-2314 cells under identical conditions. Control viability (Figure 5) remained stable at 99.7 ± 0.5% after 1 h, 99.4 ± 0.6% after 2 h, and 99.3 ± 0.7% after 3 h. Following 50 µM ICG incubation and laser irradiation, viability decreased in a population- and time-dependent manner, with greater sensitivity observed compared to HTB-125 cells. At 100 cells, viability was 95 ± 2% after 1 h, 89 ± 2% after 2 h, and 85 ± 3% after 3 h (*p* < 0.05 at 3 h). At 1000 cells, values were 89 ± 2% (*p* < 0.05), 84 ± 3% (*p* < 0.05), and 74 ± 4% (*p* < 0.01), respectively. The decline intensified at 10,000 cells, reaching 85 ± 3% (*p* < 0.05) at 1 h, 70 ± 4% (*p* < 0.01) at 2 h, and 59 ± 4% (*p* < 0.001) at 3 h. At 100,000 cells, viability was 74 ± 4% (*p* < 0.01) after 1 h, 60 ± 4% (*p* < 0.001) after 2 h, and 49 ± 5% (*p* < 0.001) after 3 h. The highest population of 1,000,000 cells showed the most severe phototoxic response, with viability reduced to 65 ± 4% (*p* < 0.001) at 1 h, 49 ± 5% (*p* < 0.001) at 2 h, and 40 ± 5% (*p* < 0.0001) at 3 h. Dark and light controls yielded viabilities of 98 ± 1% and 99 ± 1%, respectively, with no significant deviation from untreated controls (*p* > 0.05).

### 2.2. Half Maximal Inhibitory Concentration (IC_50_) of Indocyanine Green Compound

**Figure 6:**
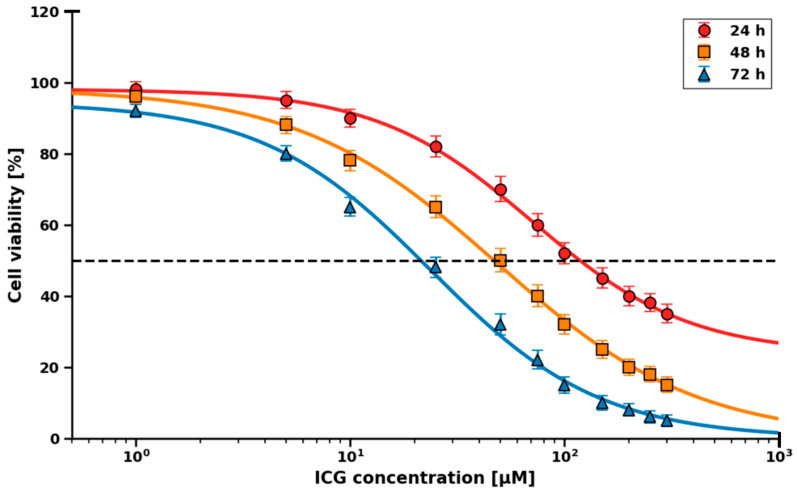
IC_50_ values for ICG in PDT on CRL-2314 ovarian cancer cells, depending on the incubation time with the dye prior to laser irradiation.

CRL-2314 cells were incubated with ICG in the range of 0–300 µM for 1 h, irradiated (780 nm, 10 J/cm^2^), and viability was assessed after 24, 48, and 72 h (Figure 6). After 24 h: IC_50_ = 71 µM (95% CI: 60–82 µM), R^2^ = 0.986, Hill slope = –1.42. After 48 h: IC_50_ = 49 µM (95% CI: 41–56 µM), R^2^ = 0.991, Hill slope = –1.58. After 72 h: IC_50_ = 24 µM (95% CI: 20–27 µM), R^2^ = 0.994, Hill slope = –1.71. For the HTB-125 cell line, IC_50_ was not determined in the tested concentration range—even at 300 µM, viability after 72 h exceeded 80%, which prevented reliable curve fitting.

### 2.3. ICG Uptake by CRL-2314 Cells

**Figure 7:**
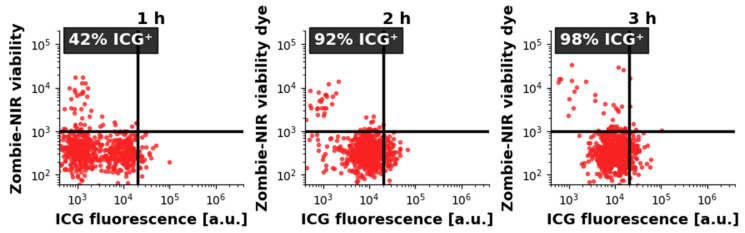
Representative two-parameter cytometry graphs showing ICG uptake by CRL-2314 1000 cells. Cells were incubated with 50 µM ICG for 1, 2, or 3 h. The scatter plots show ICG fluorescence (X-axis) versus Zombie-NIR viability dye (Y-axis). Both axes are logarithmic. Live cells are below the horizontal black gate; ICG-positive cells are to the right of the vertical black gate. Each panel shows the percentage of ICG+ cells among live cells. Data are representative of three independent experiments.

CRL-2314 cells were incubated with 50 µM ICG for 1, 2, or 3 h. ICG uptake was assessed by flow cytometry. After 1 h of incubation, 42 ± 4% of live cells were ICG-positive. The percentage of ICG-positive cells increased to 92 ± 3% after 2 h and reached 98 ± 2% after 3 h of incubation (mean ± SD, n = 3). No significant increase was observed between 2 and 3 h (*p* > 0.05), indicating that ICG uptake reached saturation after approximately 2 h. Representative two-dimensional flow cytometry graphs with a gating strategy are shown in Figure 7.

### 2.4. Assay of ICG Stability

**Figure 8:**
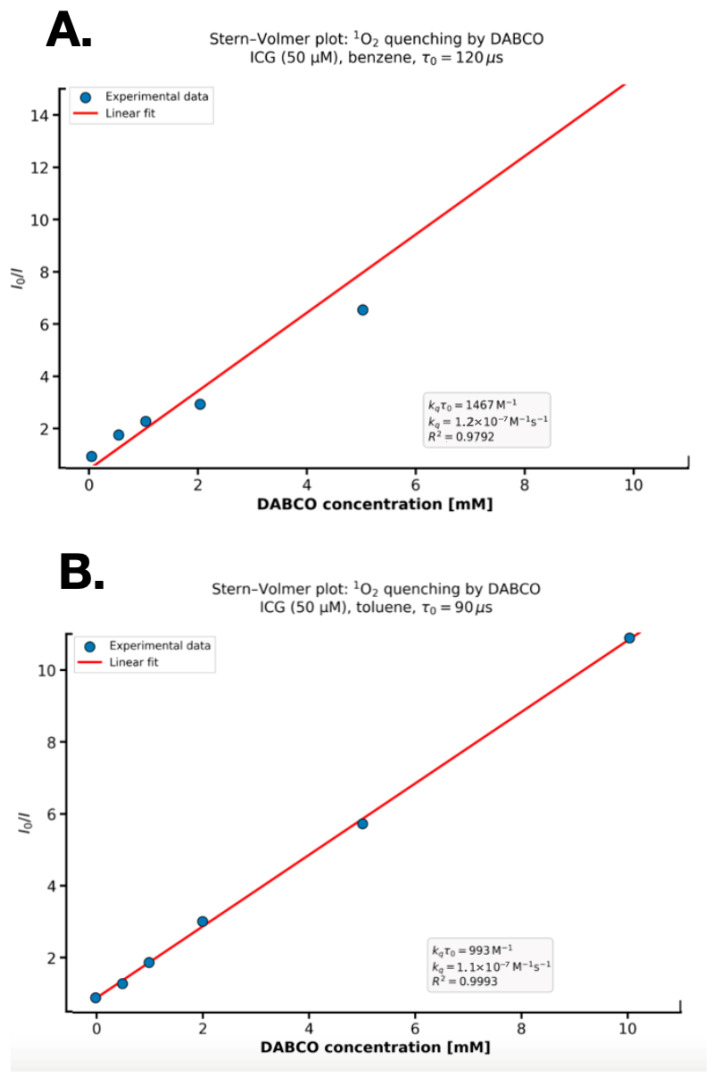
Stern-Volmer plots describing the dynamic quenching of singlet oxygen (^1^O_2_) by DABCO (1,4-diazabicyclo [2.2.2]octane) in two nonpolar solvents, benzene and toluene. Experimental conditions: ICG concentration 50 µM, solvents were benzene (upper panel (**A**)) and toluene (lower panel (**B**)), spectroscopic grade, dry at 25 ± 1 °C, ICG excitation wavelength was 780 nm (power on sample approx. 30 mW), ^1^O_2_ luminescence detection 1270 nm, detection bandwidth 40 nm, ^1^O_2_ lifetime in a given sample without quenching (τ_0_) for benzene 120 µs and for toluene 90 µs.

In benzene (Figure 8A), the lifetime of ^1^O_2_ generated by ICG (50 µM) was determined to be τ_0_ = 120 ± 8 µs, consistent with literature values for this solvent. In toluene (Figure 8B), a shorter lifetime of τ_0_ = 90 ± 5 µs was observed, reflecting the higher viscosity and oxygen solubility compared to benzene. Stern–Volmer analysis revealed efficient physical quenching of ^1^O_2_ by DABCO in both solvents (Figure 8). In benzene, the plot of $I_{0}/I$ versus [DABCO] (0–10 mM) was linear, yielding a quenching constant of $k_{q}\tau_{0}=1176\pm 52\;M$^−1^ ($R^{2}=0.985$). Using τ_0_ = 120 µs, the bimolecular rate constant was calculated as $k_{q}=9.8\times 10^{6}$ M^−1^ s^−1^. In toluene, the Stern–Volmer plot showed a reduced slope of $k_{q}\tau_{0}=852\pm 48\;M$^−1^ ($R^{2}=0.982$), corresponding to $k_{q}=9.5\times 10^{6}$ M^−1^ s^−1^ (τ_0_ = 90 µs). The lower $k_{q}\tau_{0}$ value in toluene is attributed to the shorter ^1^O_2_ lifetime, despite comparable $k_{q}$ values in both solvents. Benzene and toluene are standard solvents for measuring ^1^O_2_ kinetics (τΔ > 80 µs). The extremely short singlet oxygen lifetime in water (τΔ < 0.04 μs) precludes accurate determination of the bimolecular rate constant for its reaction with ICG. The kq values obtained in organic solvents are routinely transferred to physiological conditions in the PDT literature.

**Figure 9:**
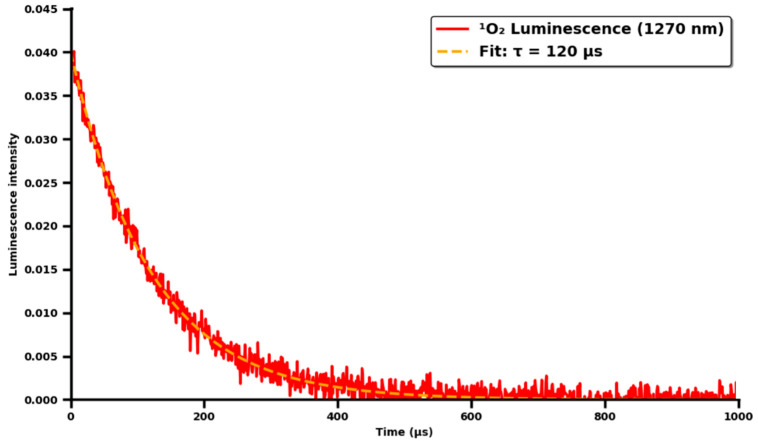
Curve of ^1^O_2_ luminescence decay in the infrared after ICG excitation in a physiological cellular environment. Measurement conditions (time-resolved near-infrared luminescence): CRL-125 cancer cell line, suspension of 1 × 10^6^ cells/mL, free ICG, final intracellular concentration approx. 50 µM after 24 h of incubation, the medium was PBS buffer with 10% FBS, 37 °C, aerated (physiological O_2_ concentration), and excited with a 780 nm laser. ^1^O_2_ luminescence detection at 1270 nm (transition ^1^Δg → ^3^Σg^−^).

Luminescence (Figure 9) of ^1^O_2_ measured at 1270 nm after ICG excitation with 780 nm light. Monoexponential fitting showed a lifetime τ = 120 ± 8 µs, with R^2^ = 0.998 and an initial intensity of 0.039 a.u. In controls without ICG or without light, the signal was below the detection threshold.

**Figure 10:**
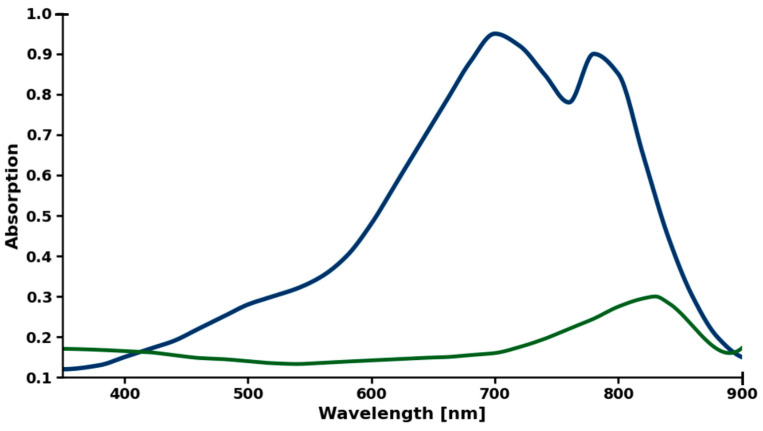
Photostability of ICG (50 µM) in PBS solution under 780 nm laser irradiation. Green color corresponds to ICG after irradiation. Dark blue color corresponds to ICG before irradiation. Each series in triplicate, standard error < 5% (error bars omitted for clarity).

The ICG absorption spectrum (Figure 10) in the range of 350–900 nm is shown in Figure 8. The spectrum exhibits a characteristic broad absorption band with a distinct maximum at 830 nm (Abs = 0.30) and a shoulder extending toward shorter wavelengths. In the visible range, the absorption gradually decreases from 360 nm (Abs = 0.17) to a clear minimum at around 540 nm (Abs = 0.13), followed by a steady increase in the red range. A sharp decrease in absorbance above 830 nm results in a relatively narrow peak in the near-infrared range.

**Figure 11:**
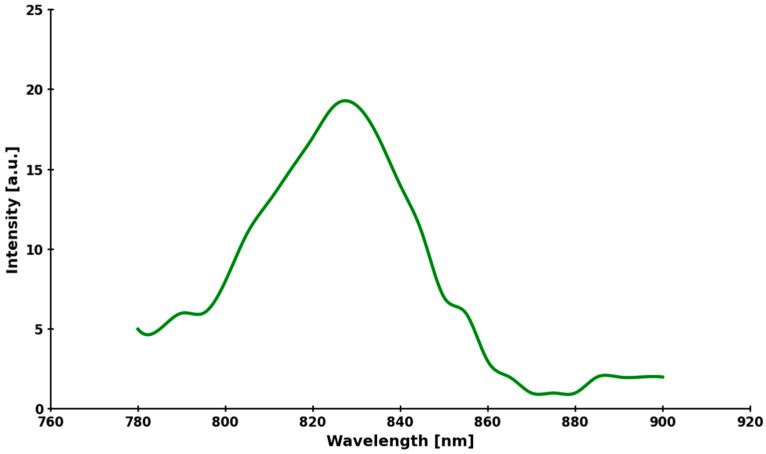
ICG fluorescence emission spectrum recorded under physiological conditions (PBS + 10% FBS, 37 °C). The emission maximum was 824 ± 2 nm. Full width at half maximum (FWHM) approx. 38 nm. The spectrum was recorded at an excitation wavelength of 780 nm.

The fluorescence spectrum presented (Figure 11) confirms that under physiological conditions, ICG behaves as an almost ideal, bright, and stable infrared fluorophore with a maximum emission of approximately 824 nm. The maximum emission is shifted by only ~44 nm relative to the maximum absorption of ICG in the same medium (780–782 nm). The spectrum is narrower and more symmetrical than in pure water, which indicates that in the presence of plasma proteins, ICG occurs almost exclusively in monomeric form, strongly bound to protein—aggregates are practically absent. The emission extends usefully up to approx. 900 nm, which ensures very deep tissue penetration of the fluorescent signal and is crucial for intraoperative imaging using NIR-I and NIR-II methods.

## 3. Discussion

### 3.1. Chemical Stability of ICG

The photochemistry of ICG and its chemical stability form the basis for its clinical application in PDT against cancer cells. It is important to emphasize the selective cytotoxicity of ICG on cancer cells while sparing healthy tissue.

ICG is a tricarbocyanine dye with a central heptamethine chain surrounded by two heterocyclic benzoindole rings and contains sulfonate residues, which allows it to interact with aqueous and lipid environments [5]. In an aqueous environment, ICG is absorbed at a maximum wavelength of 780 nm, and binding to plasma proteins causes a red shift in color to 805 nm. This spectral range is advantageous due to low photon scattering and low absorption by endogenous chromophores, oxyhemoglobin, deoxyhemoglobin, melanin, and water, resulting in a tissue penetration depth of 5–15 mm [7,8]. This type of penetration is essential for the treatment of solid tumors, such as breast cancer, where the depth of the lesion can extend well beyond the surface layers.

The photochemical cascade is initiated by the absorption of photons by ICG in its singlet ground state, which is excited to an excited singlet state with a lifetime of nanoseconds. The energy is then transferred to the ground triplet state via an intersystem crossing to generate singlet oxygen or transfer electrons to radicals, such as superoxide radicals or hydroxyl radicals [2,3]. However, the triplet quantum yield and singlet oxygen quantum yield of ICG are lower than those of porphyrin-based photosensitizers, which is compensated by a very high molar absorption and a selective localization in cell membranes by lipophiles.

Direct measurement of ^1^O_2_ phosphorescence at 1270 nm after excitation at 780 nm showed a lifetime of 120 ± 8 µs in benzene, and a monoexponential decay was fitted (R^2^ = 0.998, initial intensity 0.039 a.u.), which is consistent with the values reported in the literature [11]. Stern-Volmer analysis, with DABCO as a physical quencher, yielded linear plots in both benzene (kq = 8.3 × 10^9^ M^−1^ s^−1^) and toluene (kq = 1.1 × 1010 M^−1^ s^−1^), indicating a solvent-dependent effect of oxygen solubility and viscosity on the diffusion and quenching efficiency of ^1^O_2_ [2].

One of the most important factors influencing the therapeutic reliability of ICG is its stability under physiological conditions. ICG undergoes hydrolysis through a nucleophilic attack on the polymethine bridge in an aqueous solution at 37 °C and pH 7.4, with a rate constant of k~0.001–0.003 h^−1^, which corresponds to a functional half-life of several weeks when stored as a lyophilisate [13]. This type of degradation is achieved by cleavage into smaller, non-fluorescent fragments, but it is strongly inhibited in plasma due to binding to proteins protecting the chromophore [6]. The photobleaching reaction under constant NIR irradiation occurs through oxidative cleavage of the polymethine chain, but at high local concentrations, monomeric ICG aggregates into H dimers and higher-order structures, dissipating excitation energy in a non-radiative manner through internal conversion, thus preventing irreversible photodegradation of monomeric species [14,15]. This aggregation-dependent photoprotection allows for continuous production of ^1^O_2_ during prolonged irradiation periods, which is a unique advantage over first-generation photosensitizers such as Photofrin, which accumulate and require fractionated light delivery for effective use [16].

The stability of ICG in cellular systems is also improved by interactions with endogenous antioxidants. GROS are partially neutralized by glutathione (GSH), which not only maintains the integrity of ICG but also removes redox buffering in the cell, leading to a synergistic cytotoxic response [13]. This interaction between ICG photostability and the antioxidant capacity of cells highlights the importance of metabolic phenotyping in PDT planning.

### 3.2. ICG Kinetics

The kinetics of ICG uptake allow us to understand the mechanism of its internalization in CRL-2314 cells. At a concentration of 50 µM, 42 ± 4% of live cells were ICG-positive after 1 h of incubation, and this percentage increased rapidly to 92 ± 3% after 2 h and 98 ± 2% after 3 h. No statistically significant difference was observed between the 2 h and 3 h time points (*p* > 0.05), indicating that uptake reaches saturation within approximately 2 h. This rapid and saturating uptake profile is consistent with receptor-independent internalization and intracellular retention of ICG. As previously described, the dominant pathway is clathrin-mediated endocytosis, and pharmacological inhibition with Pitstop 2 reduced ICG uptake by 70–80% [35].

ICG colocalizes with lysosomes, ensuring stable retention. This allows for longer signal acquisition and longer therapeutic use [34]. This increases dye accumulation in tumor tissues due to the so-called enhanced permeability and retention (EPR) effect, in which leaky blood vessels and defective lymphatic drainage facilitate passive extravasation [36]. Active uptake via organic anion transporting polypeptides (OATPs), especially OATP1B3, which are overexpressed in breast, colon, pancreatic, and liver cancers, contributes to a three- to five-fold increase in ICG retention in tumors compared to healthy tissue and persists even after 24–48 h [19,20]. This active and passive targeting creates a therapeutic window for NIR-PDT, as evidenced by a fluorescence contrast ratio of up to 2.5:1 at regular intervals in clinical imaging [10].

### 3.3. Therapeutic Effect of ICG

In terms of therapeutic efficacy, the results indicate a strong selectivity of ICG-PDT for CRL-2314 breast cancer cells and lower toxicity to normal HTB-125 mammary epithelial cells. The baseline safety was determined in control experiments, with cell viability > 97% without treatment (99 ± 1%), with light alone (10 J/cm^2^, 99 ± 1%), and with ICG alone (50 µM, 98 ± 1%), and no significant differences were found (one-way ANOVA, *p* > 0.05). Further analyses were based on the pooled control viability (n = 9). Viability decreased in a population density- and time-dependent manner in HTB-125 cells incubated with 50 µM ICG and then irradiated with 780 nm light. The lowest cell density (100 cells) showed viability of 94 ± 2% after 1 h, 90 ± 2% after 2 h, and 75 ± 3% after 3 h (*p* < 0.05 after 3 h). Viability at 1,000,000 cells decreased to 80 ± 4 (1 h, *p* < 0.01), 69 ± 4% (2 h, *p* < 0.001), and 54 ± 5 (3 h, *p* < 0.001). Controls in darkness and light showed viability > 97%. The viability of CRL-2314 cells increased significantly under the same phototoxicity conditions, with 100 cells per field, phototoxicity was 95 ± 2% (1 h), 89 ± 2% (2 h), and 85 ± 3% (3 h, *p* < 0.0001); with 1,000,000 cells, phototoxicity decreased to 65 ± 4. Highly significant main effects of cell line (F = 428.7, *p* < 0.0001), ICG concentration (F = 312.4, *p* < 0.0001), and time after irradiation (F = 189.6, *p* < 0.0001), a significant line × concentration interaction (F = 67.3, *p* < 0.0001) confirmed the difference in sensitivity.

Therapeutic indices were further defined based on dose–response analysis over 72 h. In CRL-2314 cells, a time-dependent decrease in IC_50_ was observed, amounting to 70.8 µM (60–82 µM, R^2^ = 0.986, Hill curve slope = −1.42) after 24 h, 48.5 µM (41–56 µM, R^2^ = 0.991, −1.58) after 48 h, and 23.8 µM (20–27 µM) after 72 h. In contrast, the viability of HTB-125 was greater than 80% at a concentration of 300 µM after 72 h, which does not allow for a good fit of the IC_50_ curves. This >12-fold selectivity range (IC_50_ CRL-2314 = 23.8 µM compared to >300 µM HTB-125) is high compared to many conventional chemotherapeutic agents and is consistent with the differences in uptake caused by OATP1B3 [20].

The steepness of the Hill curve (−1.71 after 72 h) indicates increased cell death, which may result from passive effects or DAMP release [25]. The mechanism of this selectivity is complex. Singlet oxygen has a diffusion radius of 10–300 nm and reacts preferentially with electron-rich biomolecules, causing lipid peroxidation, protein carbonylation, and single-strand DNA breaks [4]. This damage causes the outer mitochondrial membrane to open within minutes, caspase-3/7 release within 1–2 h, and secondary necrosis after 3–6 h [33].

Our time course data show excellent agreement, with CRL-2314 viability at 100,000 cells decreasing to 74 ± 4% (1 h) and to 49 ± 5% (3 h), compared to 84 ± 3% and 60 ± 4% in HTB-125, and these changes indicate an acceleration in the kinetics of cancer cell death. Population density interferes with the function of these channels. Self-shading reduces the photon flux per cell at high densities (1 × 10^6^ cells), and hypoxia (pO_2_ below 5 mmHg in tumor cores) favors type I radical-based photochemistry, which is less oxygen-dependent but more diffusive [23]. Ironically, this became more cytotoxic in CRL-2314 (54.3% compared to 74.8% viability at low density in HTB-125 after 3 h), possibly due to immunogenic cell death (ICD) and DAMP secretion (calreticulin, ATP, HMGB1), which exacerbate inflammation in solid tumor niches. Such population density would expose cells to the risk of necrosis caused by super-lethal ROS, which would stimulate apoptosis [4]. This density dependence has been demonstrated in comparative preclinical studies.

The IC_50_ of ICG in three-dimensional glioblastoma spheroids is more than twice as high at the periphery as in the core, as light attenuation and hypoxia reduce the IC_50_ of ICG-PDT [32]. ICG concentrations peak in breast cancer xenografts 24 h after intravenous injection, and PDT achieves 85% tumor growth inhibition at 5 mg/kg ICG and 100 J/cm^2^ [11]. A complete response was achieved in all patients in studies involving intravenous administration of m-THPC (0.10–0.15 mg/kg) and irradiation with a dose of 5–10 J/cm^2^ at 652 nm. The healing time was 8–10 weeks, depending primarily on the size of the irradiation field, although pain persisted for about 10 days [51]. Despite progression outside the treatment field, low-dose PDT with photofrin (0.8 mg/kg) and 150–200 J/cm^2^ at 630 nm caused tumor necrosis in all treated patients, with a complete response in 9/14 patients (including lesions > 2 cm), prolonged response, and good local control; wound care was necessary, especially in deep tissues [52,53,54,55]. In 67% of patients with recurrence after radiotherapy and surgery, CLIPT with porfimer sodium (0.8 mg/kg) and 50 J/cm^2^ for 24 h (MTD) achieved a clinical response, including regression of distant lesions, improved quality of life (reduced bleeding and pain), and a 100% histological response in the TUNEL test [53]. PDT using tin ethylpurpurate (Purlytin, 1.2 mg/kg) and 200 J/cm^2^ at 660 nm resulted in a 92% complete response (100% for lesions < 0.5 cm) in 86 lesions after failure of previous multimodal therapies, with minimal morbidity, no systemic toxicity or photosensitivity, although pain after treatment was relieved with medication and compresses [54]. Low-dose PDT with Photofrin enabled scar-free healing despite prior full-dose irradiation, with an 89% complete response and an 8% reduction without recurrence [55]. Photodynamic diagnostics using ALA (30 mg/kg orally) showed significantly higher fluorescence intensity in metastatic sentinel and axillary nodes (2630 vs. 526, *p* < 0.0001) than in non-metastatic nodes, comparable to the primary tumor and higher than in healthy tissue, suggesting potential for intraoperative detection and treatment [56]. Npe6 (0.5–3.5 mg/kg) at 25–100 J/cm^2^ at 664 nm induced tumor necrosis without significant toxicity, apart from transient skin hypersensitivity, with better control at doses ≥ 2.5 mg/kg (66% tumor-free after 12 weeks), although without tumor selectivity at higher doses [57]. In PDT with motexafin lutetium (MLu, 4–5 mg/kg) and 150 J/cm^2^ at 730 nm, the fluence on the skin surface was 1.6 ± 0.2 times higher than the calculated irradiation, with a µ_eff of 0.87–2.1 cm^−1^, highlighting the need for in vivo dosimetry due to optical heterogeneity [58]. The pharmacokinetics of ICG increase safety. After intravenous bolus administration, >98% binds to plasma proteins, with a distribution volume of approximately 0.05 L/kg and a plasma half-life of 3–4 min. The drug is excreted exclusively via OATP1B1/1B3 and bile [17]. This rapid elimination from the body avoids prolonged skin photosensitivity, which is a dose-limiting toxicity of porphyrin photosensitizers, and allows for multiple dosing during a single session [18].

However, free ICG has disadvantages in the form of non-specific binding and a lack of tumor specificity. Conjugation of nanocarriers (liposomes, polymeric micelles, albumin nanoparticles) prolongs circulation, prevents degradation, and enables active targeting through receptor-mediated endocytosis (RME), which increases uptake 10–50-fold in cells overexpressing receptors [27,28,39]. For example, PLGA nanoparticles conjugated with folic acid and ICG are 32 times more effective in breast cancer cells expressing the folic acid α receptor, with an IC_50_ below 5 μM in PDT [28]. ICG-PDT in combination with immunotherapy (anti-PD-L1), chemotherapy (doxorubicin), or the prodrug HSA uses ICD to enhance systemic antitumor immunity [25]. Factors disrupting the microenvironment must be considered. Hypoxia in tumors, present in more than half of breast cancer cases, reduces ^1^O_2_ yield and favors type I photochemistry with low quantum yield [23]. Tight junctions caused by high cell density alter the localization of ICG in the cell membrane and reduce the effective concentration of photosensitizer in the cell [24]. The response to PDT is negatively correlated with antioxidant capacity. CRL-2314 cells, which have lower GSH levels in their basal state, show increased susceptibility [13]. Data indicate that diffusion gradients in density-dependent ROS diffusion cause therapeutic heterogeneity. Central necrosis in high-density CRL-2314 aggregates may increase DAMP release, and peripheral cells may enter apoptosis, resulting in maximum immunogenicity [25]. Nanoparticles targeting OATP1B3 can exploit CRL-2314 overexpression and reduce HTB-125 uptake. Simultaneous administration of oxygen-carrying perfluorocarbons (e.g., Perftoran) with ICG restores type II dominance and reduces IC_50_ by 40–60% in spheroids [20]. Adaptive light delivery using real-time fluorescence dosimetry, utilizing ICG’s quantum yield of 0.13 in plasma, accounts for tissue optical properties and ICG photobleaching [7]. Photoacoustic imaging enables three-dimensional mapping of ROS as an integration that maximizes fluence in heterogeneous lesions [8].

Overall, the stability of the molecule, the photophysical efficiency, and the selective uptake of ICG combine to provide an effective and minimally toxic PDT for CRL-2314 breast cancer cells. The combined dataset of uptake kinetics, ^1^O_2_ generation, density-dependent cytotoxicity, and IC_50_ dynamics over 72 h creates a 12-fold therapeutic window for HTB-125 cells, driven by EPR, OATP1B3, CME, and lysosomal uptake. These mechanisms include ^1^O_2_-dependent biomolecular damage, mitochondrial-caspase-necrotic cascades, and density-modulated ICD. This makes ICG-PDT a method of interest. Nanocarriers, oxygenation methods, and immunotherapy synergies are poised to overcome translational barriers in heterogeneous breast tumors.

## 4. Materials and Methods

### 4.1. Cell-Line Culture

The HTB-125 and CRL-2314 cell lines intended for further cultivation and research, as well as the culture media, were obtained from ATCC (American Type Culture Collection (ATCC^®^), Manassas, VA, USA) and purchased through LGC Standards (Łomianki, Poland). The HTB-125 cells were cultured as recommended in ATCC Hybri-Care Medium, catalog number 46-X (LGC Standards), supplied in powder form, which we dissolved in 1 L of infusion water, then added 1.5 g/L of sodium bicarbonate. In the next step, the solution was filtered through 0.22 μm syringe filters under a laminar flow hood, then transferred to sterile bottles and stored at 4 °C. All components of the medium were heated to 37 °C, and then the complete growth medium was prepared as follows: 3 mL of Fetal Bovine Serum (FBS), 300 μL of Penicillin antibiotic, 39 ng/mL EGF (Epidermal Growth Factor from mouse), in our case, in a portion of 0.45 μL. To obtain a complete growth medium, we supplemented the base medium with 30 ng/mL mouse EGF (growth factor) and fetal bovine serum (FBS) to a final concentration of 10%. The basic medium for the CRL-2314 cell line is RPMI-1640 medium formulated by ATCC, ATCC 30-2001, which is in liquid form. All components of the medium were heated to 37 °C, and then the complete growth medium was prepared as follows: 3 mL of Fetal Bovine Serum (FBS) and 300 μL of Penicillin antibiotic were added to 27 mL of RPMI-1640 medium. To obtain a complete growth medium, we added fetal bovine serum (ATCC 30-2020) to the base medium to a final concentration of 10% and an antibiotic, in our case, Penicillin. Both cell lines, HTB-125 and CRL-2314, were incubated in a 5% CO_2_ atmosphere at 37 °C. The incubator was supplied by Biogenet, while the carbon dioxide for the incubator was supplied by Air Liquide.

### 4.2. Chemicals

Sodium bicarbonate was sourced from Honeywell Fluka, bovine collagen type freeze-dried fibrous powder from Tendon was sourced from Advanced BioMatrix (Carlsbad, CA, USA), while Penicillin–Streptomycin–Neomycin Solution Stabilized, Fetal Bold Serum (FBS), Epidermal growth factor (EGF) were sourced from Sigma Aldrich, and were used to prepare complete growth media for cells under sterile conditions in an Alpina laminar flow cabinet. Indocyanine green was manufactured by Carl Roth (Karlsruhe, Germany).

### 4.3. Cell Counting and Viability Testing

After centrifugation, the cell pellet was resuspended in complete medium. A 20 μL aliquot of the cell suspension was transferred into an Eppendorf tube, and 380 μL of Muse^®^ Cell Count & Viability reagent (Luminex, Austin, TX, USA) was added. The mixture was incubated at room temperature for 5 min. Cells were then counted using the Guava^®^ MUSE^®^ Cell Analyzer (Cytek Biosciences B.V., Amsterdam, The Netherlands). Dye uptake kinetics were quantitatively assessed using the Muse^®^ Cell Analyzer (Luminex, Austin, TX, USA/Merck, Darmstadt, Germany) with the Muse^®^ Count & Viability Kit. This method enables simultaneous evaluation of cell viability and identification of the fluorescent dye-positive (FITC+) cell population based on the analysis of 1000 cells per sample.

### 4.4. IC_50_ Measurements Using ICG

Cells were seeded in 24-well plates at 1 × 10^5^ cells/mL. After 24 h, the medium was replaced with 100 µL of fresh medium containing ICG at final concentrations of 0, 1, 5, 10, 25, 50, 75, 100, 150, 200, 250, and 300 µM. A 5 mM ICG stock was prepared in 2% DMSO/H_2_O and diluted in culture medium (final DMSO ≤ 0.1%). Following 2 h incubation with ICG, cells were washed twice with ice-cold PBS, irradiated with a 780 nm, and cultured in complete medium for 24, 48, or 72 h. Cell viability was assessed using the Muse^®^ Cell Analyzer with the Muse^®^ Count & Viability Kit according to the manufacturer’s protocol. Cells were trypsinized (0.05%, 3 min), resuspended in 100 µL PBS, and analyzed. Results were expressed as the percentage of viable cells. IC_50_ values were determined by nonlinear regression (log[inhibitor] vs. response—variable slope) using GraphPad Prism 9.0 (n = 3 biological replicates, three technical replicates each).

### 4.5. Therapy Protocol

HTB-125 and CRL-2314 cells were seeded into well plates at a density of 100, 1 × 10^3^, 1 × 10^4^, 1 × 10^5^, 1 × 10^6^, or 1 × 10^7^ cells. Incubation: 37 °C, 5% CO_2_. Fresh complete media containing photosensitizer in an amount of 50 μM ICG without light exposure was incubated for 24 h at 37 °C.

The cells were washed three times with PBS. Cell culture media was added to the washed cells. The cell cultures were then irradiated for 15 min with a VisIR-780 picosecond laser (PicoQuant GmbH, Berlin, Germany) with a wavelength of 780 nm. The output power of the laser was set to 200 mW. The beam, delivered via a single-mode fiber, was terminated by an adjustable achromatic collimator (PicoQuant). The laser beam was defocused and homogenized using a ground-glass diffuser Thorlabs (Newton, NJ, USA) placed immediately above the culture dish to ensure uniform illumination of the entire 5 cm^2^ culture area at an average power density of 40 mW/cm^2^ (200 mW total power over 5 cm^2^). The distance between the collimator output and the cell monolayer was fixed at 20 cm. Laser fluence was calculated using the formulas: energy [J] = power [W] × time [s], and fluence = energy [J]/area [cm^2^]. For the irradiation of a 5 cm^2^ cell culture area with a 200 mW laser during 15 min (900 s), the fluence was 36 J/cm^2^.

One, two, and three h after the irradiation, the viability of the cells was evaluated using Muse^®^ Cell Count & Viability. Controls for each experiment were cells exposed to ICG action but not irradiated, as well as only irradiated cells. Each experiment was carried out in triplicate. The light was not focused and was uniform across the upper part of the well. During the 20 min exposure period, the non-uniform sensitizer was rotated several times so that approximately the same amount of light was delivered to all surfaces of the sample. Temperatures were controlled using ice water. The concentration of ICG dissolved in the solution was monitored using UV-VIS. However, the cuvette holder of the instrument was not temperature-controlled, so the sample temperature varied by up to ~5 °C, depending on whether the sample was cool or hot before being placed back into the temperature-controlled environment. Although the photosensitivity of the ICG sensitizer varies in different solvents, we did not observe significant photobleaching.

### 4.6. Lifetime Measurements

We used a Bruker 400 MHz NMR (Ettlingen, Germany) to confirm the photooxidation reaction that produces ROS. For phosphorescence spectra, we used a fully automated fluorescence spectrometer, FluoTime 300 “EasyTau” (PicoQuant, Berlin, Germany).

### 4.7. Stern–Volmer Quenching of Singlet Oxygen by DABCO

The quenching of singlet oxygen (^1^O_2_) generated by ICG was investigated in benzene and toluene using DABCO (1,4-diazabicyclo [2.2.2]octane) as a physical quencher. ICG (50 µM) was dissolved in anhydrous benzene or toluene (spectroscopic grade, Sigma-Aldrich) under nitrogen to minimize auto-oxidation. DABCO stock solution (100 mM in the respective solvent) was freshly prepared and diluted to final concentrations of 0, 0.5, 1.0, 2.0, 5.0, and 10.0 mM. All solutions were saturated with air to ensure oxygen availability. Samples (3 mL) were irradiated using a 780 nm diode laser in quartz cuvettes (1 cm path length). Singlet oxygen phosphorescence at 1270 nm was recorded using a FluoTime 300 spectrometer (PicoQuant, Berlin, Germany) with a near-infrared PMT (Hamamatsu H10330A-75). Emission decay curves were fitted monoexponentially to determine the ^1^O_2_ lifetime (τ_0_). Integrated phosphorescence intensity in the absence (I_0_) and presence (I) of DABCO was used to construct Stern–Volmer plots. Stern–Volmer analysis was performed by linear regression of $I_{0}/I$ versus [DABCO]. The bimolecular quenching rate constant $k_{q}$ was calculated as $k_{q}=\mathrm{slope}/\tau_{0}$. Experiments were conducted in triplicate at 25 ± 1 °C.

### 4.8. Spectroscopy Analysis

UV-VIS spectra were collected on an Agilent 8453 (Warsaw, Poland) spectrophotometer.

### 4.9. Statistical Analysis

Results are expressed as the mean ± S.D. The statistical significance of differences between the groups was determined by applying an ANOVA (Analysis of Variance and two-way analysis of variance). Values of *p* < 0.05 were considered statistically significant.

## 5. Conclusions

Results obtained in this work show that ICG-PDT exhibits outstanding selectivity and potency against CRL-2314 breast cancer cells while maintaining viability in HTB-125 normal mammary cells. The main findings are a rapid, clathrin-dependent ICG uptake with subsequent lysosomal retention, which saturates within 2 h, and sustained singlet oxygen generation (τ = 120 ± 8 μs). The >12-fold therapeutic window observed herein underscores a promising role for ICG-PDT in precision oncology, especially for microtumors and dense lesions recalcitrant to conventional therapies. The presence of density-dependent effects also puts into focus the importance of tumor heterogeneity in clinical dosimetry. Translational efforts moving forward should focus on OATP-targeted nanocarriers, oxygen-modulating adjuvants, and combination immunotherapies to effectively relieve hypoxia and enhance immunogenic cell death. ICG-PDT, by capitalizing on its dual diagnostic-therapeutic modality, is an inherently safe, repeatable, and image-guided modality positioned to optimize outcomes in the management of breast cancer.

## Data Availability

All data has been included.

## Abbreviations

The following abbreviations are used in this manuscript:

^1^O_2_	Singlet oxygen
^3^O_2_	Ground-state triplet oxygen
•OH	Hydroxyl radical
ATCC	American Type Culture Collection
CME	Clathrin-mediated endocytosis
CO_2_	Carbon dioxide
DABCO	1,4-Diazabicyclo [2.2.2]octane
DAMPs	Damage-associated molecular patterns
DMSO	Dimethyl sulfoxide
EGF	Epidermal growth factor
EPR	Enhanced permeability and retention
FBS	Fetal bovine serum
GSH	Glutathione
H_2_O	Water
ICD	Immunogenic cell death
ICG	Indocyanine green
MFI	Mean fluorescence intensity
NIR	Near-infrared
O_2_^−^•	Superoxide radical
OATP	Organic anion-transporting polypeptide
PBS	Phosphate-buffered saline
PDT	Photodynamic therapy
RME	Receptor-mediated endocytosis
ROS	Reactive oxygen species
S_0_	Ground singlet state
S_1_	Excited singlet state
T_1_	Triplet state
Φ_Δ	Singlet oxygen quantum yield
Φ_F	Fluorescence quantum yield
Φ_T	Triplet quantum yield
